# Genetic alterations and protein expression in combined small cell lung cancers and small cell lung cancers arising from lung adenocarcinomas after therapy with tyrosine kinase inhibitors

**DOI:** 10.18632/oncotarget.9083

**Published:** 2016-04-28

**Authors:** Xiaohua Shi, Huanli Duan, Xuguang Liu, Liangrui Zhou, Zhiyong Liang

**Affiliations:** ^1^ Department of Pathology, Peking Union Medical College Hospital, Chinese Academy of Medical Science, Beijing, China

**Keywords:** combined small cell lung cancer, adenocarcinoma, epidermal growth factor receptor, KRAS, retinoblastoma protein

## Abstract

There are 2 hypotheses regarding the mechanism underlying the adenocarcinoma (AD) to small cell lung cancer (SCLC) transition in patients receiving Tyrosine kinase inhibitor (TKI) therapy: 1) AD gives rise to SCLC owing to the pressure of the TKI therapy, and 2) the SCLC coexists with the AD de novo, but is not detected in biopsy specimens of the heterogeneous tumor. In this study, we try to address this issue by examination the genetic alteration and protein expression profile between SCLC arising from AD, and SCLC in combined small cell lung cancers (CSCLC). In the former, the SCLC had the same genetic profile as the AD, and we strongly suggest that the transition was a consequence of TKI therapy. In the latter, genetic alterations and protein expression tended to differ between the NSCLC and SCLC components of the CSCLC. The results showed that *EGFR* and *KRAS* mutation were found in 1 but not both component of CSCLC, and the NSCLC component usually expressed the EGFR and RB1 proteins, whereas the SCLC component did not. This finding indicates that the NSCLC and SCLC components arose separately and that CSCLC are unsuitable for TKI therapy despite the presence of sensitive *EGFR* mutations.

## INTRODUCTION

Small cell lung cancers (SCLC), which account for 15% of lung cancers, differ histologically and biologically from non-small cell lung cancers (NSCLC), which account for 85% of all lung cancers [1]. Combined SCLC (CSCLC) is a mixture of SCLC and NSCLC, in which the NSCLC component can be adenocarcinoma (AD), squamous cell carcinoma (SCC), or large cell carcinoma. CSCLC is rare, accounting for only 2% to 10% of SCLC [2, 3]. The treatment strategies for SCLC and NSCLC differs: concurrent chemotherapy and radiation therapy for most SCLC, and neoadjuvant chemotherapy and complete resection for early-stage NSCLC [4]. There is still no well-established treatment for CSCLC because of its rarity and complexity.

The introduction of tyrosine kinase inhibitors (TKI) dramatically has greatly improved the treatment of AD with *EGFR* mutations. However, acquired resistance to TKI usually develops after about 12 months of TKI treatment [5]. There are several mechanisms for the development of resistance, one of them being the histological transformation from AD to SCLC, as confirmed by repeated biopsy of the SCLC [6]. Whether the SCLC component exists before TKI treatment or is a consequence of treatment is controversial.

It is generally accepted that NSCLC originates in the bronchoalveolar junction or in the basal cells of the bronchial membrane, whereas SCLC originates in the neuroendocrine cells underneath the basal membrane of the bronchi [7]. The origin of CSCLC is unclear; the two components may give rise to each other or simply coexist in a single tumor.

In this study, we reported 11 cases of CSCLC and 2 cases of SCLC that arose from AD after TKI treatment. We focused on the clinicopathological, immunohistochemical, and genetic profiles of the distinct components of these tumors to determine their origin.

## RESULTS

### Clinical characteristics of the patients

Clinical characteristics of the patients are summarized in Table 1. Thirteen patients were divided into 4 groups. Group 1 contained two patients with SCLC originating from AD owing to acquired resistance after TKI therapy. Group 2 contained four patients with CSCLC, whose NSCLC components were AD. Group 3 contained five patients with CSCLC, whose NSCLC component was SCC. Group 4 contained two patients with CSCLC, whose NSCLC component were SCC and AD.

**Table 1:** Clinical characteristics of the patients

Group	Case #	Sex	Age (years)	Smoking	TKI	Biopsy or Surgery	Final Stage	Recur	RFS (months)	Death	OS (months)
1 AD to SCLC	1–1	M	46	N	Y	B	IV	Y	10	Y	21.3
	1–2	F	48	N	Y	B	II	Y	31	N	139.6
2 SCLC + AD	2–1	M	79	Y	N	S	III	Y	19.2	N	19.2
	2–2	M	71	Y	N	S	I	Y	11	Y	12
	2–3	M	66	Y	N	S	III	Y	13	Y	14.4
	2–4	M	61	Y	N	B	IV	Y	12	Y	12.8
3 SCLC + SCC	3–1	M	71	Y	N	S	NA	Y	5	N	71.4
	3–2	M	62	Y	N	S	III	N	-	N	35.5
	3–3	M	57	Y	N	B	IV	Y	25	Y	24.8
	3–4	M	66	Y	N	S	I	Y	6	N	8.7
	3–5	M	74	Y	N	B	NA	Y	18	Y	18
4 SCLC + AD + SCC	4–1	M	66	NA	NA	S	NA	NA	NA	NA	NA
	4–2	M	58	Y	N	S	II	N	-	Y	6.3

TKI: tyrosine kinase inhibitor; Recur: recurrence; RFS: recurrence-free survival; OS: overall survival; AD: adenocarcinoma; SCLC: small cell lung cancer; SCC: squamous cell carcinoma; F: female; M: male; Y: yes; N: no; NA: not available; B: biopsy; S: surgery.

The ages of the two patients in Group 1 were 46 and 48 years old, respectively; case 1 was a male and case 2 was a female, and neither had a smoking history. In case 1, the patient was diagnosed with stage IV AD and had received TKI (gefitinib) therapy. The patient obtained stable disease (SD) for 10 months and died 11 months after being diagnosed with SCLC. In case 2, the patient was diagnosed with stage II AD and received surgery followed by chemotherapy. Recurrence was noticed after a follow-up period of 31 months. TKI therapy was then administrated, resulting in SD of 61 months. Then she had repeated biopsy of the lesion, which was found to have transformed to SCLC. Her treatment was consequently switched to chemotherapy and radiotherapy. She is now surviving with the tumor. The follow-up time is 139.6 months. In both patients, the SCLC was located at a different site from the AD, and re-biopsy results confirmed the diagnosis.

The average age of the eleven patients with CSCLC (groups 2–4) was 66.5 years (range: 57–79 years); all were men, 10 were smokers, and none had received TKI therapy. Clinical stage was determined according to the pathological and radiological information: two patients at stage I, one at stage II, three at stage III, and two at stage IV. The stages were not determined for the remaining three patients. Because their disease was advanced. three patients were biopsied only. Seven patients underwent pneumonectomy. One patient had thoracotomy, but the surgery did not proceed owing to the diagnosis of CSCLC (based on the result of frozen sections) and the advanced stage of the disease. Seven of the eight surgical patients received postoperative chemotherapy or radiation after being informed the benefits and risks of the treatments; one patient refused further therapy. Recurrence was noticed in eight of ten patients at averagely13.9 months after diagnosis. Six of the ten patients died at averagely 14.7 months after diagnosis. At the end of the follow-up period, one patient was alive without disease, and two were alive with disease.

### Pathological characteristics

**Figure 1:**
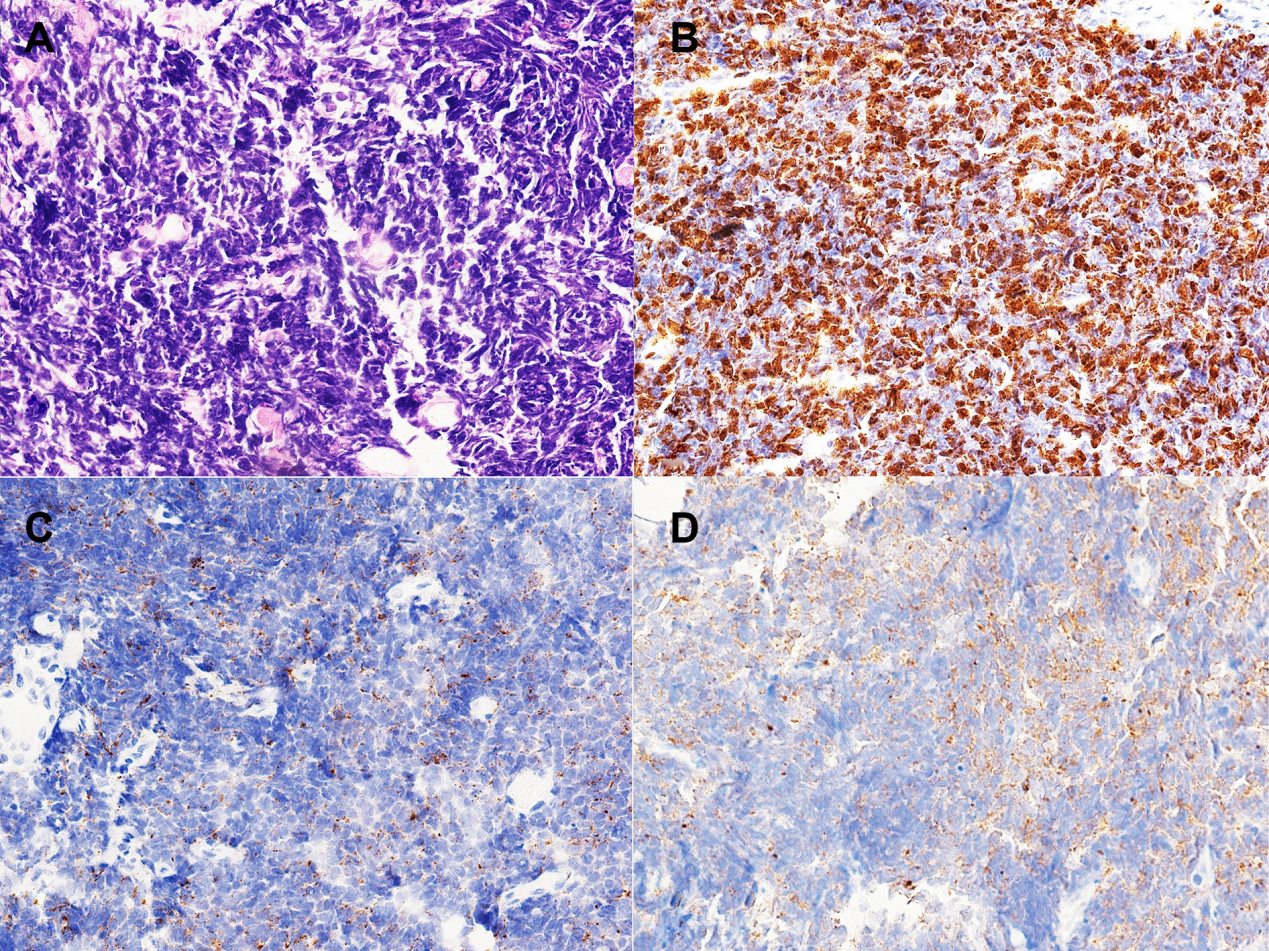
Morphology and immunohistochemistry of small cell lung carcinoma (SCLC) (**A**) Morphology of SCLC (×40); (**B**) Ki-67 index is about 90% in SCLC (×40); (**C**) Syn immunostain is positive in SCLC. (×40); (**D**) CgA immunostain is positive in SCLC. (×40).

The pathological characteristics of the patients are presented in Table 2. The SCLC component in all cases was confirmed by immunohistochemistry (IHC), which showed positive staining of chromogranin A (CgA), Synaptophysin (Syn), and a high proliferation index (high percentage of Ki-67-positive cells) (Figure 1).

**Table 2:** The pathological characteristics of the patients

Group	Case #	Separate	Tumor Size (cm)			AD pattern	SCC Differentiation	Node	Invol of Bronch	Invol of Pleura	LVI
			AD	SCLC	SCC						
1	1–1	Y	5	1.5		Acinar	-	-	-	-	-
	1–2	Y	2.3	NA	-	Acinar	-	-	-	-	-
2	2–1	Y	1.5	3.5	-	Micropap	-	Y	N	N	Y
	2–2	Y	1.8	1.8	-	Lepidic	-	-	-	N	Y
	2–3	N	3.5, 30%	70%	-	Acinar	-	Y	N	Y	Y
	2–4	N	NA	NA	-	Acinar	-	-	-	-	-
3	3–1	Y	-	0.7	2.8	-	Poor	-	-	Y	N
	3–2	Y	-	1.2	5	-	Moderate	Y	Y	Y	Y
	3–3	Y	-	3.5	2.9	-	Well	-	-	-	-
	3–4	N	-	6 20%	80%	-	Well	N	Y	N	Y
	3–5	N	-	NA	NA	-	Moderate	-	-	-	-
4	4–1	N	2.6 2.5%	2.5%	95%	Solid	Poor	-	-	N	N
	4–2	Y	5%*	2.1	4.5 95%	Acinar	Well	N	Y	Y	Y

AD: adenocarcinoma; SCLC: small cell lung cancer; SCC: squamous cell carcinoma; Invol of Bronch: involvement of the bronchus; Invol of Pleura: involvement of the pleura; LVI: lymphovascular invasion; Y: yes; N: no; NA: not available; Micropap: micropapillary.

* The AD component is mixed with SCC component.

Among the eleven CSCLC cases (groups 2–4), six had distinguishable SCLC and NSCLC components. The average size of the SCLC component was 2.1 cm (range: 0.7–3.5 cm), while the average size of the NSCLC component was 3.1 cm (range: 1.5–5 cm). In three of the eleven cases in which the SCLC and NSCLC component were mixed together, the ratio of the two different components was estimated according to the morphology of the HE-stained samples. The most common growth pattern of the AD component was the alveolar pattern (three cases); other patterns included micropapillary (one case), solid (one case), and lepidic (one case). The differentiation status of the SCC component was rated as well- (three cases), moderately- (two cases), or poorly-differentiated (two cases). Positive node metastasis was observed in three of five cases; the SCLC components metastasized in two cases, and metastases of both the SCLC and AD components were observed in one case. Three of five cases showed involvement of the major bronchi, while four of eight cases showed involvement of the pleura. Lymphovascular invasion was identified in six of eight cases.

### Immunohistochemical results and genetic profiles

The immunohistochemical and genetic profiling results are summarized in Table 3.

**Table 3:** The immunohistochemical and genetic profiling results of the patients

Item	Component	Group 1		Group 2				Group 3					Group 4	
		1–1	1–2	2–1	2–2	2–3	2–4	3–1	3–2	3–3	3–4	3–5	4–1	4–2
p53	AD	NA	NA	1	0	1	1						1	1
	SCLC	0	NA	0	1	1	0	1	1	1	1	0	1	1
	SCC							0	0	1	1	0	1	1
RB1	AD	NA	NA	0	0	1	1						1	0
	SCLC	0	NA	0	0	0	0	0	1	0	0	0	1	1
	SCC							1	1	NA	1	1	1	1
EGFR IHC	AD	NA	1	1	1	1	1						1	1
	SCLC	0	NA	0	0	0	0	0	0	1	0	0	0	0
	SCC							1	1	1	0	1	1	1
*EGFR* mutation	AD	E21 L858R	E19 DEL	WT	E21 L858R	WT	WT						WT	WT
	SCLC	E21 L858R	E19 DEL	WT	WT	WT	WT	WT	WT	WT	WT	WT	WT	WT
	SCC							WT	WT	WT	WT	WT	WT	WT
*KRAS* mutation	AD	WT	WT	WT	WT	WT	WT						WT	WT
	SCLC	WT	WT	WT	WT	WT	WT	WT	E2 G12V	WT	WT	WT	WT	WT
	SCC							WT	WT	WT	WT	WT	WT	WT

IHC: immunohistochemistry, RB1: retinoblastoma 1; EGFR: epidermal growth factor receptor; AD: adenocarcinoma; SCLC: small cell lung cancer; SCC: squamous cell carcinoma; NA: not available; WT: wild type; E: exon; 0: negative; 1: positive.

In Group 1, the primary AD and recurrent SCLC had identical *EGFR* mutations (a L858R mutation in exon 21 in case 1 and an exon 19 deletion in case 2). In the CSCLC cases (groups 2–4), 2 mutations were identified : an *EGFR* mutation (L858R in exon 21) in the AD component of case 2 in Group 2, and a *KRAS* mutation (G12V in exon 2) in the SCLC component of case 2 in Group 3. No mutation in *BRAF*, *ALK*, or *PIK3CA* was found in any of the cases in Group 1–4.

In Group 1, immunohistochemical results were unavailable for the AD component in case 1 and for both component in case 2 because of limited tissue amount or quality. In case 1, RB1, p53, and EGFR were not expressed in the SCLC component, while in case 2, *EGFR* was amplified in the AD component.

In groups 2–4 (the eleven CSCLC cases), p53 was not expressed in the SCLC component in three cases or in the NSCLC component in four cases. RB1 expression was negative in the SCLC component in eight cases, but was negative in the NSCLC component in only three cases. EGFR expression was positive in the NSCLC components in ten cases, but was positive in the SCLC component in only one case.

In summary, the same kind of *EGFR* mutations was observed in both the AD and SCLC in Group 1. In contrast, there were differences in the gene mutation profiles and protein expression patterns in the SCLC and NSCLC components of the CSCLC (groups 2–4): Divergent genetic changes were noted in the SCLC and NSCLC component in two cases (EGFR mutation only in the AD component of case 2 in Group 2, and K-ras mutation only in the SCLC component of case 2 in Group 3). EGFR was amplified more often in the NSCLC component, whereas loss of RB1 was more common in the SCLC component.

## DISCUSSION

The present study reported the clinicopathological, immunohistochemical, and genetic characteristics of two SCLC cases that arose from AD after TKI therapy and eleven CSCLC cases. Our results showed differences between the “transitioned” tumors (AD to SCLC) and the combined tumors (CSCLC): the former often had the same *EGFR* mutations before and after the transition, while the latter expressed RB1 and EGFR in the NSCLC component more frequently than that in the SCLC component, *EGFR* and *KRAS* mutation was found in 1 but not both components of CSCLC.

In our study, all 11 patients with CSCLC were male smokers, which was consistent with the work of Lu et al. [8]. *EGFR* mutations were identified in one patient with combined SCLC and AD, but in zero patients with combined SCLC and SCC. Such mutation rate is much lower than that for lung AD in Asian populations, but is consistent with the data reported for CSCLC [9]. Table 4 summarizes the eight previously published cases of CSCLC with *EGFR* mutations, and here, we added an additional case. In the eight previous cases, the average age was 64 years; four patients were women and four were men. There were more smokers compared to non-smokers (five versus three). In Asian populations, young non-smoking women diagnosed with AD are likely to have an *EGFR* mutation unique to them [12]. This is not the case for CSCLC, as was shown in Table 4. In our study, the patients in Group 1 (AD recurring as SCLC) were young nonsmokers, so it was with AD patients that harbored *EGFR* mutations.

**Table 4:** CSCLC with EGFR mutation in review of literature

Author	Year	NSCLC Component	Age (years)	Sex	Smoking	EGFR Mutation	EGFR Amplication
Fukui et al. [9]	2007	AD	62	F	N	E21 L858R in both	NA
Tatematsu et al. [10]	2008	AD	69	M	Y	E21 L858R in both	Amp in AD
		AD	65	M	Y	E19 DEL in both	Amp in AD
Lu et al. [8]	2012	AD	62	F	N	E19 DEL in both	NA
		SCC	61	M	Y	**E19 DEL in SCLC**	NA
Norkowski et al. [11]	2013	AD	62	M	N	E18 G719A and 21 DEL in both	NA
		AD	66	F	Y	**E19 DEL in AD**	NA
		AD	65	F	Y	**E21 L858R in AD**	NA

NSCLC: non small cell lung cancer; AD: adenocarcinoma; SCC: squamous cell carcinoma; F: female; M: male; DEL: deletion; NA: not available; Amp: amplication.

There are two hypotheses regarding the mechanism underlying the AD to SCLC transition in patients receiving TKI therapy: 1) AD gives rise to SCLC owing to the pressure of the TKI therapy, and 2) the SCLC coexists with the AD de novo, but is not detected in biopsy specimens of the heterogeneous tumor [13]. Table 5 summarizes the 18 previously published cases in which AD recurred as SCLC after TKI therapy. This summary shows that the AD and SCLC have identical *EGFR* mutations in most cases, which is in concordance with the two cases described in our study. This observation prompted us to investigate the genetic status of CSCLC, especially those with an AD component. Only a few studies have examined the genetic differences between the AD and SCLC components of CSCLC, and most of them focused on a single gene (e.g., *EGFR*) [8, 9]. In our study, we used a lung cancer gene panel to assess multiple genetic alterations, not only in four CSCLC with SCLC and AD components, but also in five CSCLC with SCLC and SCC components. The results of our analysis showed genetic disparities between SCLC and AD or SCC, most notably in terms of *EGFR* and *KRAS* mutations. As shown in Table 4, three of the eight cases of CSCLC with known *EGFR* mutations also showed different genetic alterations in these components. Taken together, the present study showed that SCLC arising from AD was different from the SCLC component of CSCLC in terms of their genetic profiles. These results suggested that SCLC originated from AD because of TKI therapy, whereas the two components of CSCLC arised separately, and form a blended tumor. Although the clear mechanism of transition from AD to SCLC remains unknown, it is believed that the existence of a pluripotent stem cell population and the AD phenotype can switch to SCLC under the positive selection of EGFR TKI [9, 17].

**Table 5:** AD recurred as SCLC after TKI therapy in review of literature

Author	Year	Age	Sex	Smoking	TKI	EGFR Mutation in AD	EGFR Mutation in SCLC	Others
Zakowski et al. [14]	2006	45	F	N	Y	NA	19 DEL	
Morinaga et al. [15]	2007	46	F	N	Y	19 DEL	19 DEL	
Alam et al. [16]	2008	73	F	N	Y	L858R	L858R	
Tatematsu et al. [10]	2008	36	F	N	Y	L858R	L858R	
Sequist et al. [17]	2011	67	F	N	Y	L858R	L858R	
		54	F	NA	Y	19 DEL	19 DEL	
		56	F	NA	Y	L858R	L858R	PIK3CA in SCLC
		40	F	NA	Y	19 DEL	19 DEL	
		61	F	NA	Y	L858R	L858R	
Ma et al. [18]	2012	65	F	N	Y	L858R	L858R	
Van Riel et al. [19]	2012	42	F	N	Y	19 DEL	19 DEL	T790M in AD after TKI
Popat et al. [20]	2013	46	F	N	Y	19 DEL	20 DEL	Large cell NEC identified on resistance
Watanabe et al. [6]	2013	52	F	N	Y	19 DEL	19 DEL	
Norkowski et al. [11]	2013	60	F	N	Y	19 DEL	21 E872K	
		50	F	N	Y	19 DEL	19 DEL	
Facchinettir et al. [21]	2013	74	F	N	Y	L858R	L858R	
Hwang et al. [22]	2015	61	M	Y	Y	19 DEL	19 DEL	
Furugen et al. [23]	2015	63	M	N	Y	19 DEL	19 DEL	T790M in AD after TKI in autopsy

TKI: thyrosine kinase therapy; AD: adenocarcinoma; SCLC: small cell lung cancer; F: female; M: male; DEL: deletion; NA: not available; NEC: neuroendocrine carcinoma.

In addition to genetic differences, protein expression differences detected by IHC were noticed in the two components of the CSCLC. Our results showed that down regulation of RB1 protein expression was more common in the SCLC component than the NSCLC component of CSCLC. The loss of the *RB1* gene is observed in almost all cases of SCLC, including one case in which the SCLC was derived from an AD. This result suggested that the mutation or loss of *RB1* could be a characteristic feature of SCLC [24]. *EGFR* mutations have also been found in SCLC; however, SCLC with *EGFR* mutations is less responsive to TKI therapy than lung AD with *EGFR* mutations. Although SCLC arising from AD after TKI therapy harbors the same *EGFR* mutations before and after transition, *EGFR* amplification is often lower in the SCLC than in the AD, which may account for the worse response of SCLC to TKI therapy [24]. In our study, IHC was used to assess EGFR protein expression in the 2 components of the CSCLC. Our results showed that EGFR was not frequently expressed in the SCLC component (negative results in ten of the eleven cases). This finding indicated that TKI might not be suitable for CSCLC treatment, despite the presence of *EGFR* mutations.

The present study is subjected to the following limitations. First, because both AD to SCLC transition and CSCLC are rare conditions, we were unable to enroll sufficient cases in the present study to demonstrate a statistical significance. Second, while we have sought to provide extra evidence to support our hypotheses that TKI induced AD to SCLC transition and SCLC and NSCLC arise separately in CSCLC by including previously reported cases in the discussion section, we were unable to draw a definitive conclusion without more substantial evidences. Given the limitations mentioned above, we hope to increase our sample size in the future and look for more direct evidences to further prove the conclusions.

In summary, there were clinical, immunohistochemical, and genetic differences between SCLC arising from AD, and CSCLC. First, our results of identical EGFR mutation in AD and SCLC supported that the transition was a consequence of TKI therapy. Secondly, genetic alterations and protein expression were different between the NSCLC and SCLC components of the CSCLC. Specifically, *EGFR* was found only in the AD component of one CSCLC and *KRAS* mutation was identified in the SCLC component of another CSCLC. The NSCLC component usually expressed the EGFR and RB1 proteins more often than the SCLC component in CSCLC. ALL these finding supported that CSCLC is a collision tumor and it was unsuitable for TKI therapy despite the presence of *EGFR* mutations.

## MATERIALS AND METHODS

### Patients

Thirteen patients with a final diagnosis of CSCLC who were biopsied or underwent surgery at Peking Union Medical College Hospital in Beijing, China between January 2010 and December 2014 were enrolled in this study. Among these patients, 2 had an AD that transformed into an SCLC after TKI therapy, and 11 had CSCLC. All final diagnoses were based on the morphology of tumor samples stained with hematoxylin and eosin (HE), and confirmed by immunohistochemistry (IHC) and a review of the HE-stained samples by 2 pathologists, individually.

Clinical information was extracted from a digital or archival database and included patient age, sex, and smoking habits (never-smoker was defined as less than 100 cigarettes lifetime), clinical stages, postoperative treatment methods, and prognosis. Clinical stages were determined at the time of surgery based on the tumor-node-metastasis staging system of the 7th edition of the American Joint Commission on Cancer. Recurrence free survival (RFS) was defined as the time from surgery to relapse or the conclusion of the study. Overall survival (OS) was calculated as the time from surgery to death or the conclusion of the research.

In cases involving surgical procedures, pathological characteristics (e.g., tumor location, involvement of the pleura, lymphovascular invasion, and node metastasis) were collected. This study was approved by the Institutional Review Board of the Peking Union Medical College Hospital.

### Immunohistochemistry

Formalin-fixed, paraffin-embedded tissue samples with a thickness of 4 μm were used for IHC. Staining was performed through using a Ventana Benchmark XT autostainer (Ventana Medical Systems Inc., Tucson, AZ, USA) according to the manufacturer's instructions. The proteins examined and the antibodies used included the following: retinoblastoma protein 1 (RB1; 1:50, polyclonal; Abcam, Cambridge, UK), p53 (1:1, monoclonal; MXB, Beijing, China), EGFR (1:500, polyclonal; Roche, Tucson, USA), chromogranin A (CgA; 1:100, polyclonal; OriGene, Beijing, China), synapsin (Syn; 1:500, polyclonal; DAKO, Carpinteria, CA, USA), Ki-67 (1:500, monoclonal; OriGene), thyroid transcription factor receptor-1 (TTF-1; 1:1, monoclonal; MXB), and p40 (1:500, polyclonal; DAKO). The scoring system used for human epidermal growth factor receptor-2 evaluation was used for EGFR evaluation. In this system, expression is graded from 0–3+ according to the percentage and intensity of positive cells; 0 and 1 indicate negative expression, and 2 and 3 indicate positive expression. RB1, p53, Ki-67, p40, and TTF-1 were scored as positive if brown nuclear staining was evident. CgA, and Syn were scored as positive if there was brown staining in the cytoplasm.

### Genetic alterations

The NSCLC and SCLC components of the CSCLC that could be distinguished in HE-stained samples were manually macrodissected by a pathologist. DNA was isolated from paraffin-embedded tissue by using a QIAamp DNA FFPE Tissue Kit (Qiagen, Hilden, Germany). DNA concentration was measured by using a Nanodrop 2000 spectrophotometer (ThermoFisher, Wilmington, DE, USA) and further standardized to 20–50 ng/μL.

The mutation profiles of the *EGFR*, *KRAS*, *PIK3CA*, and *BRAF* genes were determined by using the appropriate human gene mutation detection kit from Beijing ACCB Biotech (Beijing, China). Sixty-three hotspot mutations were examined: 45 in exons 18–21 of *EGFR*, 12 in exons 2 and 3 of *KRAS*, 5 in exons 9 and 20 of *PIK3CA*, and the *BRAF* V600E mutation. Quantitative polymerase chain reaction (PCR) was performed by using an Mx3000P PCR system (Agilent, Santa Clara, CA, USA) with the following settings: 95°C for 10 minutes, 40 cycles at 95°C for 15 seconds, and 60°C for 1 minute. Results were interpreted as recommended by the manufacturer.

Mutations were detected by next generation sequencing (NGS). The Ion Torrent system (Life Technologies) was used to analyze NGS libraries prepared by the NextDaySeq-lung Cancer Library Preparation Panel Kit (Beijing ACCB Biotech). Briefly, pooled primers were used to amplify the genomic regions of the exons noted above, followed by ligation with adapters and barcodes. After purification, libraries were quantified by a Qubit dsDNA HS Assay Kit and a Qubit 2.0 fluorimeter (Life Technologies, Carlsbad, CA, USA), diluted to a concentration of 3 ng/mL, and pooled in equal volume. The pooled library was clonally amplified via emulsion PCR using ion sphere particles, and templatepositive particles were enriched by an Ion OneTouch 2 system (Life Technologies) as specified by the manufacturer. After enrichment, sequencing primers and the polymerase were added (PGM Sequencing Supplies 200 v2 Kit; Life Technologies). The libraries were loaded onto an ion 318 chip (Life Technologies) and sequenced by an Ion Torrent system. Variants were identified and annotated by a proprietary DanPA bioinformatics pipeline (Beijing ACCB Biotech).

As a confirmative method, Sanger sequencing was performed. Genomic regions of *EGFR* exons 18–21, *KRAS* exons 2 and 3, *PIK3CA* exons 9 and 20, and *BRAF* exons 11 and 15 were amplified from the DNA samples. Each exon was sequenced bidirectionally using the same primers as used in the initial amplification reaction and the ABI Prism Big Dye Terminator v 3.1 Cycle Sequencing Kit (Applied Biosystems, Foster City, CA, USA). The results of the sequencing primer extension reactions were analyzed by an ABI 3130XL Genetic Analyzer (Applied Biosystems) according to the manufacturer's instructions.

The three methods were carried out in all specimen except the AD of case 2 in Group 1 because the limitation of tissue quality, only PCR was done to examine the mutation status of *EGFR, KRAS, PIK3CA*, and *BRAF*.
